# An algorithm of calculating transport parameters of thermoelectric materials using single Kane band model with Riemann integral methods

**DOI:** 10.1038/s41598-022-09734-4

**Published:** 2022-04-29

**Authors:** Fei-Hung Lin, Chia-Jyi Liu

**Affiliations:** Department of Physics, National Changhua University of Education, Changhua, 500 Taiwan

**Keywords:** Energy science and technology, Materials science, Mathematics and computing

## Abstract

We develop an algorithm called SKBcal to conveniently calculate within minutes the thermoelectric transport parameters such as reduced Fermi level (*η*), electronic thermal conductivity (*κ*_*e*_), lattice thermal conductivity (*κ*_*l*_), Hall factor (*A*), effective mass (m^*^), quality factor (*β*) and theoretical *zT* within the framework of single Kane band (SKB) model. The generalized Fermi–Dirac integral for SKB model is integrated by left Riemann integral method. A concept of significant digits of relative error is involved to determine the accuracy of calculation. Furthermore, a combined program of *"For"* and *"While"* is coded to set up an iteration for refining the reduced Fermi level. To easily obtain the quality factor, we re-derive the expression into a formula related to carrier mobility. The results calculated by SKBcal are consistent with the data reported in the literatures.

## Introduction

One can analyze transport properties of a given thermoelectric material using the single parabolic band (SPB) for conduction in a single parabolic band^1^ or the single Kane band (SKB) model for conduction in a single nonparabolic band. Either model can be used to calculate parameters of the reduced Fermi level (*η*), electronic thermal conductivity (*κ*_*e*_), density-of-states effective mass at the band edge (*m*_0_^*^) and other transport parameters by using experimentally determined thermopower and Hall concentration. One could therefore use the carrier concentration (*n*) dependence of the quality factor (*β*), which is calculated using the above models as a strategy to optimize the dimensionless figure of merit *zT* = *σS*^*2*^*/κ*, where *σ*, *S*, and *κ* are the electrical conductivity, thermopower, and thermal conductivity, respectively.

For the energy band theory in solids, the crystal momentum dependence of the energy would be nonparabolic if the direct energy gap is small at the appropriate band edge or the carrier concentration is high. Kane found a highly nonparabolic band in InSb and good agreement between experimental data and the calculated results on fundamental absorption and its dependence on *n*-type impurity concentration^2^.

To begin with, the reduced Fermi energy is first calculated by using experimentally determined thermopower (Seebeck coefficient) and two Fermi–Dirac integrals. The Lorenz number is then calculated by three Fermi–Dirac integrals using the as-obtained reduced Fermi energy. As seen in the following equations^3,4^,

1 $$S = \pm \frac{{k_{B} }}{e}\left( {\frac{{ ^{1} F_{ - 2}^{1} }}{{ ^{0} F_{ - 2}^{1} }} - \eta } \right),$$

2 $$L = \left( {\frac{{k_{B} }}{e}} \right)^{2} \left[ {\frac{{ ^{2} F_{ - 2}^{1} }}{{ ^{0} F_{ - 2}^{1} }} - \left( {\frac{{ ^{1} F_{ - 2}^{1} }}{{ ^{0} F_{ - 2}^{1} }}} \right)^{2} } \right],$$

3 $$^{n} F_{k}^{m} = \mathop \int \limits_{0}^{\infty } \left( { - \frac{\partial f}{{\partial x}}} \right)x^{n} \left( {x + \varepsilon x^{2} } \right)^{m} \left[ {\left( {1 + 2\varepsilon x} \right)^{2} + 2} \right]^{k/2} dx,$$

where *S* is the thermopower, *k*_*B*_ the Boltzmann constant, *e* the elementary charge, *η* the reduced Fermi level, *L* the Lorenz number, $^{n} F_{k}^{m}$ the Fermi–Dirac integral for the SKB model, *f* the Fermi distribution, *ε* = *k*_*B*_*T/E*_*g*_ the reciprocal reduced band gap, and *x* = 

*E/k*_*B*_*T* the reduced energy. The Fermi–Dirac integral in Eq. (3) appears to be a little complex and will be discussed later. For the ± sign in Eq. (1), the positive and negative signs refer to *p*-type and *n*-type materials, respectively. Moreover, the electronic thermal conductivity (*κ*_*e*_) is calculated using the Wiedemann–Franz law as shown in Eq. (4). The lattice thermal conductivity would be obtained by subtracting the experimentally determined total thermal conductivity from the calculated electronic thermal conductivity using Eq. (5),

4 $$\kappa_{e} = L\sigma T$$

5 $$\kappa_{l} = \kappa - \kappa_{e} .$$

The Hall factor (*A*) is calculated by solving the Boltzmann transport equation using approximations of relaxation time and magnetic field. It can be expressed as the ratio of the conductivity effective mass to the density-of-states effective mass, which is usually rewritten in a simpler form^5^,

6 $$A_{i} = \frac{{3K\left( {K + 2} \right)}}{{\left( {2K + 1} \right)^{2} }} ,$$

where *K* is the ratio of the longitudinal to the transverse effective mass as given in Eq. (7). The term of *K* represents a factor indicating the band structure anisotropy, which is assumed as unity (isotropic) for simplicity in calculation^6^. In the SKB model, the Hall factor is given by Eq. (8)^4^,

7 $$K = m_{||}^{*} /m_{ \bot }^{*}$$

8 $$A = \frac{{3K\left( {K + 2} \right)}}{{\left( {2K + 1} \right)^{2} }}\frac{{ ^{0} F_{ - 4}^{1/2} \; ^{0} F_{0}^{3/2} }}{{\left( { ^{0} F_{ - 2}^{1} } \right)^{2} }}$$

To obtain the density-of-states effective mass (*m*_0_^*^) at the band edge, chemical carrier concentration (*n*) should be first calculated by multiplying the Hall concentration (*n*_*H*_) and the Hall factor together as given by Eq. (9). According to the expression for carrier concentration given in Eq. (10), *m*_0_^*^ can be derived and calculated using Eqs. (10) and (11)^3,4,6,7^,

9 $$n_{H} = \frac{1}{{eR_{H} }} = \frac{n}{A}$$

10 $$n = \frac{{\left( {2m_{0}^{*} k_{B} T} \right)^{3/2} }}{{3\pi^{2} \hbar^{3} }} {^{0}F_{0}^{3/2}}$$

11 $$m_{0}^{*} = \frac{{\hbar^{2} }}{{2k_{B} T}}\left( {\frac{{3n\pi^{2} }}{{ ^{0} F_{0}^{3/2} }}} \right)^{2/3} .$$

The Hall mobility (*μ*_*H*_) is given by Eq. (12),

12 $$\mu_{H} = A\frac{{6\pi \hbar^{4} eC_{l} }}{{\left( {2m_{b}^{*} k_{B} T} \right)^{3/2} m_{I}^{*} E_{def}^{2} }}\frac{{ ^{0} F_{ - 2}^{1} }}{{ ^{0} F_{0}^{3/2} }},$$

where $\hbar$is the reduced Planck constant, *e* the elementary charge, *C*_*l*_ a combination of some elastic moduli relevant to the acoustical vibrations^3,8^, *m*_b_^*^ the average band mass (density-of-states effective mass for each band pocket or valley)^4,6^, *m*_I_^*^ the inertial mass of the carriers along the conducting direction (inertial effective mass)^4,9^, and *E*_*def*_ the deformation potential coefficient^3,4,6–8^. Moreover, the relation between density-of-states effective mass for each band pocket and *m*_0_^*^ is given as^4^,

13 $$m_{0}^{*} = N_{v}^{2/3} m_{b}^{*} ,$$

where the *N*_*v*_ is the number of degenerate carrier pockets^4,6,7^. Therefore, Eq. (12) can be rewritten as the following,

14 $$\mu_{H} = A\frac{{6\pi \hbar^{4} eC_{l} N_{v} }}{{\left( {2m_{0}^{*} k_{B} T} \right)^{3/2} m_{I}^{*} E_{def}^{2} }}\frac{{ ^{0} F_{ - 2}^{1} }}{{ ^{0} F_{0}^{3/2} }}.$$

Finally, the theoretical *zT* can be estimated using Eq. (15),

15 $$zT = \frac{{\left( {\frac{{ ^{1} F_{ - 2}^{1} }}{{ ^{0} F_{ - 2}^{1} }} - \eta } \right)^{2} }}{{\left[ {\frac{{ ^{2} F_{ - 2}^{1} }}{{ ^{0} F_{ - 2}^{1} }} - \left( {\frac{{ ^{1} F_{ - 2}^{1} }}{{ ^{0} F_{ - 2}^{1} }}} \right)^{2} } \right] + \left( {3\beta ^{0} F_{ - 2}^{1} } \right)^{ - 1} }} ,$$

where *β* is the quality factor of thermoelectrics and can be expressed as Eq. (16),

16 $$\beta = \frac{{2k_{B}^{2} T\hbar C_{l} N_{v} }}{{3\pi m_{I}^{*} E_{def}^{2} \kappa_{l} }}.$$

Chasmar and Stratton first described that the maximum *zT* could be determined by the quality factor^10^. As seen in Eq. (16), it can be readily seen that the combination of the lager number of band valley (*N*_*v*_), small inertial effective mass (*m*_I_^*^), and low lattice thermal conductivity (*κ*_*l*_) is beneficial to obtain high-performance thermoelectric materials. It can also be used as one of the strategies to select or engineer a good thermoelectric material.

The SKB model has been adopted to estimate the material parameters and optimize the thermoelectric performance for several material systems such as InSb, PbTe, PbSe, PbS, SnTe, SnS, Cu_2_SnSe_4_, ZrNiSn, and elemental Te^2–4,6,7,11–18^, which indicates the importance of the SKB model in understanding thermoelectric materials with nonparabolic model, especially for chalcogenide materials. However, the knowledge of band gap (*E*_*g*_), the band degeneracy (*N*_*v*_), and the ratio of longitudinal to transverse effective mass (*K*) of the material are required to calculate the thermoelectric parameters using the SKB model.

The Fermi–Dirac integral for the SKB model seems a little complex than that of the SPB model and difficult to compute; it cannot be easily carried out by some mathematical software. However, the calculation can be realized using approximate values with mathematical methods. In this work, we calculate the Fermi–Dirac integral with a simple mathematical method of Riemann integral using software programs written in Python. Furthermore, we calculate the parameters of the reduced Fermi level (*η*), Lorenz number (*L*), electronic thermal conductivity (*κ*_*e*_), lattice thermal conductivity (*κ*_*l*_), Hall factor (*A*), carrier concentration (*n*), carrier mobility (*μ*), density-of-states effective mass (*m*_0_^*^) at the band edge, quality factor (*β*), and dimensionless figure of merit (*zT*), which are then saved into a file with the CSV file format. In Riemann method, the accuracy can be determined by the partition size for each subinterval. The relation between the partition size and the significant digits of relative error (SDORE) of some calculated parameters would be discussed.

## Mathematics

### Derivation of generalized Fermi–Dirac integral for SKB model

Zawadzki et al*.* derived a general formula for generalized Fermi–Dirac integral (GFDI) from the integrals of properties of semiconductors with non-parabolic spherical energy bands as expressed in Eq. (17)^19^,

17 $$^{n} L_{k}^{m} = \mathop \int \limits_{0}^{\infty } \left( { - \frac{\partial f}{{\partial x}}} \right)x^{n} \left( {x + \varepsilon x^{2} } \right)^{m} \left( {1 + 2\varepsilon x} \right)^{k} dx,$$

where $^{n} L_{k}^{m}$is a general formula of GFDI, *f* the Fermi distribution, *ε* = *k*_*B*_*T/E*_*g*_ the reciprocal reduced band gap, and *x* = *E/k*_*B*_*T* the reduced energy. In the model of calculating transport properties, the relaxation time must be introduced in the integral function. Thus, Eq. (17) should be rewritten as a complete GFDI for the SKB model given by^3^,

18 $$^{n} R_{k}^{m} = \mathop \int \limits_{0}^{\infty } \frac{{\left( { - \frac{\partial f}{{\partial x}}} \right)x^{n} \left( {x + \varepsilon x^{2} } \right)^{m} \left( {1 + 2\varepsilon x} \right)^{k} }}{{\left[ {\tau_{\left( x \right)}^{2} \left( {x + \varepsilon x^{2} } \right)\left( {1 + 2\varepsilon x} \right)^{2} } \right]^{k/4} }}dx,$$

where $^{n} R_{k}^{m}$is the complete GFDI for the SKB model, *f* the Fermi distribution, *ε* = *k*_*B*_*T/E*_*g*_ the reciprocal reduced band gap, and *x* = *E/k*_*B*_*T* the reduced energy. Besides, *τ*_*(x)*_ is the relaxation time for the acoustic phonon scattering and can be expressed as

19 $$\frac{1}{{\tau_{\left( x \right)} }} = \frac{{\pi k_{B} TE_{def}^{2} }}{{\hbar C_{l} N_{v} }}D_{\left( x \right)} \left[ {1 - \frac{{8\varepsilon \left( {x + \varepsilon x^{2} } \right)}}{{3\left( {1 + 2\varepsilon x} \right)^{2} }}} \right],$$

where *D*_*(x)*_ is the density of state as a function of *x*. In general, the density of states in SKB model is considered as a function of energy and is given by^3^,

20 $$D_{\left( E \right)} = \frac{{\sqrt 2 m_{0}^{*3/2} }}{{\pi^{2} \hbar^{3} }}E^{1/2} \left( {1 + 2\frac{E}{{E_{g} }}} \right)\left( {1 + \frac{E}{{E_{g} }}} \right)^{1/2} .$$

where *m*_0_^*^ is the density-of-states effective mass at the band edge, $\hbar$the reduced Plank constant, *E*_*g*_ the band gap, and *E* the energy. In order to combine both the variables of relaxation time and density of state, the definitions of the reciprocal reduced band gap (*ε* = *k*_*B*_*T/E*_*g*_) and the reduced energy (*x* = *E/k*_*B*_*T*) should be substituted into the expression of the density of state. Thus, the density of states can be rewritten as

21 $$D_{\left( x \right)} = \frac{{\sqrt {2k_{B} T} m_{0}^{*3/2} }}{{\pi^{2} \hbar^{3} }}\left( {x + \varepsilon x^{2} } \right)^{1/2} \left( {1 + 2\varepsilon x} \right).$$

Now, the relaxation time can be further rewritten as

22 $$\frac{1}{{\tau_{\left( x \right)} }} = \frac{{\sqrt 2 \left( {k_{B} T} \right)^{3/2} m_{0}^{*3/2} E_{def}^{2} }}{{\pi \hbar^{4} C_{l} N_{v} }}\left( {x + \varepsilon x^{2} } \right)^{1/2} \left( {1 + 2\varepsilon x} \right)\left[ {1 - \frac{{8\varepsilon \left( {x + \varepsilon x^{2} } \right)}}{{3\left( {1 + 2\varepsilon x} \right)^{2} }}} \right].$$

After simplification, we would obtain a simplified formula given by

23 $$\frac{1}{{\tau_{\left( x \right)} }} = \frac{{\left( {2k_{B} Tm_{0}^{*} } \right)^{3/2} E_{def}^{2} }}{{6\pi \hbar^{4} C_{l} N_{v} }}\left( {x + \varepsilon x^{2} } \right)^{1/2} \left( {1 + 2\varepsilon x} \right)^{ - 1} \left[ {\left( {1 + 2\varepsilon x} \right)^{2} + 2} \right].$$

In fact, the total relaxation time consists of two components: acoustic phonon scattering and optical phonon scattering. The combined formula is expressed as

24 $$\frac{1}{{\tau_{total} }} = \frac{1}{{\tau_{acoustic} }} + \frac{1}{{\tau_{optical} }}.$$

In the calculation of transport parameters using the SKBcal algorithm, we assume the acoustic phonon scattering is dominant within the framework of the SKB model. Therefore, the symbol of *τ*_*(x)*_ would represent the relaxation time for scattering by acoustic phonons or the total relaxation time in this work.

Now, we can substitute the expression of relaxation time into Eq. (18) and obtain

25 $$^{n} R_{k}^{m} = \left[ {\frac{{\left( {2k_{B} Tm_{0}^{*} } \right)^{3/2} E_{def}^{2} }}{{6\pi \hbar^{4} C_{l} N_{v} }}} \right]^{k/2} \mathop \int \limits_{0}^{\infty } \begin{array}{*{20}c} {\left( { - \frac{\partial f}{{\partial x}}} \right)x^{n} \left( {x + \varepsilon x^{2} } \right)^{m} } \\ {\left[ {\left( {1 + 2\varepsilon x} \right)^{2} + 2} \right]^{k/2} dx} \\ \end{array} .$$

There is a constant term preceding the integral and is only dependent on the index *k*. Thus, we can ignore the constant term in some parameter calculations, such as the reduced Fermi level, Lorenz number, Hall factor, and carrier concentration. However, the expression of Hall mobility has two GFDIs of one with the index *k* = -2 in numerator and the other with *k* = 0 in denominator. For convenience, the constant term in Hall mobility was taken out of the numerator part in GFDI, which is already shown in Eq. (14). Then, the form of GFDI with the SKB model in Eq. (26) can be adopted to calculate all of the parameters in this study. Here we repeat the Eq. (26) for a convenient reading,

26 $$^{n} F_{k}^{m} = \mathop \int \limits_{0}^{\infty } \left( { - \frac{\partial f}{{\partial x}}} \right)x^{n} \left( {x + \varepsilon x^{2} } \right)^{m} \left[ {\left( {1 + 2\varepsilon x} \right)^{2} + 2} \right]^{k/2} dx.$$

### Derivation of transport parameters within the framework of SKB model

In this section, our derivation of the formulas for transport parameters will involve a simplified Kane’s band model^20^, which is also called first order nonparabolicity^21^. As seen in the shape function,

27 $$\gamma \left( E \right) = \mathop \sum \limits_{n = 0}^{k} \gamma_{n + 1} E^{n + 1} ,$$

the nonparabolicity of energy band is determined by the index *k*. The band structure is parabolic if *k* is 0. In order to describe the non-parabolic band model, only the first two terms in Eq. (27) are used,

28 $$\gamma \left( E \right) = \gamma_{1} E + \gamma_{2} E^{2} ,$$

where *γ*_*1*_ is unity due to the parabolic band. Substituting Eq. (28) into the definition of density-of-states effective mass,

29 $$m^{*} = m_{0}^{*} \frac{d\gamma }{{dE}} = m_{0}^{*} \left( {1 + 2\gamma_{2} E} \right),$$

where *γ*_*2*_ is the reciprocal band gap. One can also obtain this relation by using a simplified Kane’s model given by,

30 $$\frac{{\hbar^{2} k^{2} }}{{2m_{b}^{*} }} = E\left( {1 + \frac{E}{{E_{g} }}} \right).$$

Equation (30) shows the dependence of the wave number on the energy. Besides, the effective mass (m*) entering into the transport properties of carries for a spherical energy band of arbitrary shape has the energy dependence of

31 $$\frac{{\hbar^{2} k}}{{m^{*} }} = \frac{dE}{{dk}}.$$

Using Eq. (30) and Eq. (31), the relation between m* and m_0_^*^ is given by

32 $$m^{*} = m_{b}^{*} \left( {1 + 2\frac{E}{{E_{g} }}} \right),$$

which is equivalent to Eq. (29). In general, an integral is defined as

33 $$A = \mathop \int \limits_{0}^{\infty } \left( { - \frac{\partial f}{{\partial E}}} \right)Ak^{3} dE$$

for deriving the transport parameters. Furthermore, the average values are defined as

34 $$\overline{A} = \frac{A}{1}$$

With the above definitions and assumptions, the carrier concentration in the band is given by

35 $$n = \frac{1}{{3\pi^{2} }}1.$$

By substituting Eq. (30) and Eq. (33) into Eq. (35), the expression of the carrier concentration can be rewritten as

36 $$\begin{aligned} n & = \frac{1}{{3\pi^{2} }}\left( {\frac{{2m_{0}^{*} k_{B} T}}{{\hbar^{2} }}} \right)^{3/2} \mathop \int \limits_{0}^{\infty } \left( { - \frac{\partial f}{{\partial E}}} \right)\left( {E + \frac{{E^{2} }}{{E_{g} }}} \right)^{3/2} dE \\ & = \frac{1}{{3\pi^{2} }}\left( {\frac{{2m_{0}^{*} k_{B} T}}{{\hbar^{2} }}} \right)^{3/2} \mathop \int \limits_{0}^{\infty } \left( { - \frac{\partial f}{{\partial x}}} \right)\left( {x + \varepsilon x^{2} } \right)^{3/2} dx \\ & = \frac{{\left( {2m_{0}^{*} k_{B} T} \right)^{3/2} }}{{3\pi^{2} \hbar^{3} }} {^{0} F_{0}^{3/2}} , \\ \end{aligned}$$

where *ε* = *k*_*B*_*T/E*_*g*_ is the reciprocal reduced band gap and *x* = *E/k*_*B*_*T* the reduced energy. Furthermore, the expression of the carrier mobility is given by

37 $$\begin{aligned} \mu & = \frac{e}{{m^{*} }}\tau_{\left( x \right)} \\ & = \frac{{6\pi \hbar^{4} eC_{l} N_{v} }}{{\left( {2m_{0}^{*} k_{B} T} \right)^{3/2} m_{b}^{*} E_{def}^{2} }}\frac{1}{{\left( {x + \varepsilon x^{2} } \right)^{1/2} \left[ {\left( {1 + 2\varepsilon x} \right)^{2} + 2} \right]}}, \\ \end{aligned}$$

where *τ*_*(x)*_ is the relaxation time for the acoustic scattering process. The average of carrier mobility is then expressed as

38 $$\begin{aligned} \overline{\mu } & = \frac{\mu }{1} = \frac{{\mathop \int \nolimits_{0}^{\infty } \left( { - \frac{\partial f}{{\partial E}}} \right)\frac{e}{{m^{*} }}\tau_{\left( x \right)} k^{3} dE}}{{\mathop \int \nolimits_{0}^{\infty } \left( { - \frac{\partial f}{{\partial E}}} \right)\tau_{\left( x \right)} k^{3} dE}} \\ & = \frac{{6\pi \hbar^{4} eC_{l} N_{v} }}{{\left( {2m_{0}^{*} k_{B} T} \right)^{3/2} m_{b}^{*} E_{def}^{2} }}\frac{{\mathop \int \nolimits_{0}^{\infty } \left( { - \frac{\partial f}{{\partial x}}} \right)\left( {x + \varepsilon x^{2} } \right)^{1} \left[ {\left( {1 + 2\varepsilon x} \right)^{2} + 2} \right]^{ - 2/2} dx}}{{\mathop \int \nolimits_{0}^{\infty } \left( { - \frac{\partial f}{{\partial x}}} \right)\left( {x + \varepsilon x^{2} } \right)^{3/2} dx}} \\ & = \frac{{6\pi \hbar^{4} eC_{l} N_{v} }}{{\left( {2m_{0}^{*} k_{B} T} \right)^{3/2} m_{b}^{*} E_{def}^{2} }}\frac{{ ^{0} F_{ - 2}^{1} }}{{ ^{0} F_{0}^{3/2} }}. \\ \end{aligned}$$

As compared to Eq. (14), it can be seen that the inertial effective mass (*m*_I_^*^) is equivalent to the average band mass (*m*_b_^*^). The contributions of scattering by charged impurity centers and polar optical phonons are ignored in the calculation within the framework of the SKB model. Besides, we do not consider the anisotropic materials. Hence, the equality between the two effective masses might arise from only considering the acoustic scattering events and isotropic material. Moreover, the Hall factor, *A,* which is used to convert the Hall mobility and Hall concentration into charge carrier mobility, is the product of anisotropy factor (*A*_*k*_) and statistical factor (*A*_*τ*_) and is given by^3^

39 $$\begin{aligned} A & = A_{k} A_{\tau } = \frac{{3K\left( {K + 2} \right)}}{{\left( {2K + 1} \right)^{2} }}\frac{{\overline{{\mu^{2} }} }}{{\overline{\mu }^{2} }} \\ & = \frac{{3K\left( {K + 2} \right)}}{{\left( {2K + 1} \right)^{2} }}{{\left\{ {\frac{{\mathop \int \nolimits_{0}^{\infty } \left( { - \frac{\partial f}{{\partial E}}} \right)\left[ {\frac{e}{{m^{*} }}\tau_{\left( x \right)} } \right]^{2} k^{3} dE}}{{\mathop \int \nolimits_{0}^{\infty } \left( { - \frac{\partial f}{{\partial E}}} \right)k^{3} dE}}} \right\}} \mathord{\left/ {\vphantom {{\left\{ {\frac{{\mathop \int \nolimits_{0}^{\infty } \left( { - \frac{\partial f}{{\partial E}}} \right)\left[ {\frac{e}{{m^{*} }}\tau_{\left( x \right)} } \right]^{2} k^{3} dE}}{{\mathop \int \nolimits_{0}^{\infty } \left( { - \frac{\partial f}{{\partial E}}} \right)k^{3} dE}}} \right\}} {\left[ {\frac{{\mathop \int \nolimits_{0}^{\infty } \left( { - \frac{\partial f}{{\partial E}}} \right)\frac{e}{{m^{*} }}\tau_{\left( x \right)} k^{3} dE}}{{\mathop \int \nolimits_{0}^{\infty } \left( { - \frac{\partial f}{{\partial E}}} \right)k^{3} dE}}} \right]^{2} }}} \right. \kern-\nulldelimiterspace} {\left[ {\frac{{\mathop \int \nolimits_{0}^{\infty } \left( { - \frac{\partial f}{{\partial E}}} \right)\frac{e}{{m^{*} }}\tau_{\left( x \right)} k^{3} dE}}{{\mathop \int \nolimits_{0}^{\infty } \left( { - \frac{\partial f}{{\partial E}}} \right)k^{3} dE}}} \right]^{2} }} \\ & = \frac{{3K\left( {K + 2} \right)}}{{\left( {2K + 1} \right)^{2} }}\frac{{\mathop \int \nolimits_{0}^{\infty } \left( { - \frac{\partial f}{{\partial x}}} \right)\left( {x + \varepsilon x^{2} } \right)^{1/2} \left[ {\left( {1 + 2\varepsilon x} \right)^{2} + 2} \right]^{ - 4/2} dx}}{{\left\{ {\mathop \int \nolimits_{0}^{\infty } \left( { - \frac{\partial f}{{\partial x}}} \right)\left( {x + \varepsilon x^{2} } \right)^{1} \left[ {\left( {1 + 2\varepsilon x} \right)^{2} + 2} \right]^{ - 2/2} dx} \right\}^{2} }}\frac{{\left[ {\mathop \int \nolimits_{0}^{\infty } \left( { - \frac{\partial f}{{\partial x}}} \right)\left( {x + \varepsilon x^{2} } \right)^{3/2} dx} \right]^{2} }}{{\mathop \int \nolimits_{0}^{\infty } \left( { - \frac{\partial f}{{\partial x}}} \right)\left( {x + \varepsilon x^{2} } \right)^{3/2} dx}} \\ & = \frac{{3K\left( {K + 2} \right)}}{{\left( {2K + 1} \right)^{2} }}\frac{{ ^{0} F_{ - 4}^{1/2} \;^{0} F_{0}^{3/2} }}{{\left( { ^{0} F_{ - 2}^{1} } \right)^{2} }} \\ \end{aligned}$$

For thermoelectric properties, the thermopower without the presence of magnetic field is given by

40 $$\begin{aligned} S & = \pm \frac{{k_{B} }}{e}\left[ {\frac{x\mu }{\mu } - \eta } \right] = \pm \frac{{k_{B} }}{e}\left[ {\frac{{\mathop \int \nolimits_{0}^{\infty } \left( { - \frac{\partial f}{{\partial E}}} \right)x\frac{e}{{m^{*} }}\tau_{\left( x \right)} k^{3} dE}}{{\mathop \int \nolimits_{0}^{\infty } \left( { - \frac{\partial f}{{\partial E}}} \right)\frac{e}{{m^{*} }}\tau_{\left( x \right)} k^{3} dE}} - \eta } \right] \\ & = \pm \frac{{k_{B} }}{e}\left[ {\frac{{\mathop \int \nolimits_{0}^{\infty } \left( { - \frac{\partial f}{{\partial x}}} \right)x\frac{1}{{\left( {x + \varepsilon x^{2} } \right)^{1/2} \left[ {\left( {1 + 2\varepsilon x} \right)^{2} + 2} \right]}}\left( {x + \varepsilon x^{2} } \right)^{3/2} dx}}{{\mathop \int \nolimits_{0}^{\infty } \left( { - \frac{\partial f}{{\partial x}}} \right)\frac{1}{{\left( {x + \varepsilon x^{2} } \right)^{1/2} \left[ {\left( {1 + 2\varepsilon x} \right)^{2} + 2} \right]}}\left( {x + \varepsilon x^{2} } \right)^{3/2} dx}} - \eta } \right] \\ & = \pm \frac{{k_{B} }}{e}\left[ {\frac{{\mathop \int \nolimits_{0}^{\infty } \left( { - \frac{\partial f}{{\partial x}}} \right)x^{1} \left( {x + \varepsilon x^{2} } \right)^{1} \left[ {\left( {1 + 2\varepsilon x} \right)^{2} + 2} \right]^{ - 2/2} dx}}{{\mathop \int \nolimits_{0}^{\infty } \left( { - \frac{\partial f}{{\partial x}}} \right)\left( {x + \varepsilon x^{2} } \right)^{1} \left[ {\left( {1 + 2\varepsilon x} \right)^{2} + 2} \right]^{ - 2/2} dx}} - \eta } \right] = \pm \frac{{k_{B} }}{e}\left( {\frac{{ ^{1} F_{ - 2}^{1} }}{{ ^{0} F_{ - 2}^{1} }} - \eta } \right) \\ \end{aligned}$$

where the plus sign in ± is for hole carriers and minus sign for electron carriers. Furthermore, the Lorenz number in the Wiedemann–Franz law used for calculating electronic thermal conductivity and estimating the lattice thermal conductivity is expressed as^22^

41 $$\begin{aligned} L & = \left( {\frac{{k_{B} }}{e}} \right)^{2} \left[ {\frac{{x^{2} \mu }}{\mu } - \frac{{x\mu^{2} }}{{\mu^{2} }}} \right] \\ & = \left( {\frac{{k_{B} }}{e}} \right)^{2} \left[ {\frac{{\mathop \int \nolimits_{0}^{\infty } \left( { - \frac{\partial f}{{\partial E}}} \right)x^{2} \frac{e}{{m^{*} }}\tau_{\left( x \right)} k^{3} dE}}{{\mathop \int \nolimits_{0}^{\infty } \left( { - \frac{\partial f}{{\partial E}}} \right)\frac{e}{{m^{*} }}\tau_{\left( x \right)} k^{3} dE}} - \frac{{\left( {\mathop \int \nolimits_{0}^{\infty } \left( { - \frac{\partial f}{{\partial E}}} \right)x\frac{e}{{m^{*} }}\tau_{\left( x \right)} k^{3} dE} \right)^{2} }}{{\left( {\mathop \int \nolimits_{0}^{\infty } \left( { - \frac{\partial f}{{\partial E}}} \right)\frac{e}{{m^{*} }}\tau_{\left( x \right)} k^{3} dE} \right)^{2} }}} \right] \\ & = \left( {\frac{{k_{B} }}{e}} \right)^{2} \left[ {\frac{{\mathop \int \nolimits_{0}^{\infty } \left( { - \frac{\partial f}{{\partial x}}} \right)\frac{{x^{2} \left( {x + \varepsilon x^{2} } \right)^{3/2} }}{{\left( {x + \varepsilon x^{2} } \right)^{1/2} \left[ {\left( {1 + 2\varepsilon x} \right)^{2} + 2} \right]}}dx}}{{\mathop \int \nolimits_{0}^{\infty } \left( { - \frac{\partial f}{{\partial x}}} \right)\frac{{\left( {x + \varepsilon x^{2} } \right)^{3/2} }}{{\left( {x + \varepsilon x^{2} } \right)^{1/2} \left[ {\left( {1 + 2\varepsilon x} \right)^{2} + 2} \right]}}dx}}} \right. \\ & \quad \left. { - \frac{{\left( {\mathop \int \nolimits_{0}^{\infty } \left( { - \frac{\partial f}{{\partial x}}} \right)\frac{{x\left( {x + \varepsilon x^{2} } \right)^{3/2} }}{{\left( {x + \varepsilon x^{2} } \right)^{1/2} \left[ {\left( {1 + 2\varepsilon x} \right)^{2} + 2} \right]}}dx} \right)^{2} }}{{\left( {\mathop \int \nolimits_{0}^{\infty } \left( { - \frac{\partial f}{{\partial x}}} \right)\frac{{\left( {x + \varepsilon x^{2} } \right)^{3/2} }}{{\left( {x + \varepsilon x^{2} } \right)^{1/2} \left[ {\left( {1 + 2\varepsilon x} \right)^{2} + 2} \right]}}dx} \right)^{2} }}} \right] \\ & = \left( {\frac{{k_{B} }}{e}} \right)^{2} \left[ {\frac{{\mathop \int \nolimits_{0}^{\infty } \left( { - \frac{\partial f}{{\partial x}}} \right)\frac{{x^{2} \left( {x + \varepsilon x^{2} } \right)^{1} }}{{\left[ {\left( {1 + 2\varepsilon x} \right)^{2} + 2} \right]^{2/2} }}dx}}{{\mathop \int \nolimits_{0}^{\infty } \left( { - \frac{\partial f}{{\partial x}}} \right)\frac{{\left( {x + \varepsilon x^{2} } \right)^{1} }}{{\left[ {\left( {1 + 2\varepsilon x} \right)^{2} + 2} \right]^{2/2} }}dx}} - \frac{{\left( {\mathop \int \nolimits_{0}^{\infty } \left( { - \frac{\partial f}{{\partial x}}} \right)\frac{{x\left( {x + \varepsilon x^{2} } \right)^{1} }}{{\left[ {\left( {1 + 2\varepsilon x} \right)^{2} + 2} \right]^{2/2} }}dx} \right)^{2} }}{{\left( {\mathop \int \nolimits_{0}^{\infty } \left( { - \frac{\partial f}{{\partial x}}} \right)\frac{{\left( {x + \varepsilon x^{2} } \right)^{1} }}{{\left[ {\left( {1 + 2\varepsilon x} \right)^{2} + 2} \right]^{2/2} }}dx} \right)^{2} }}} \right] \\ & = \left( {\frac{{k_{B} }}{e}} \right)^{2} \left[ {\frac{{ ^{2} F_{ - 2}^{1} }}{{ ^{0} F_{ - 2}^{1} }} - \left( {\frac{{ ^{1} F_{ - 2}^{1} }}{{ ^{0} F_{ - 2}^{1} }}} \right)^{2} } \right] \\ \end{aligned}$$

Finally, the dimensionless figure of merit *zT* is expressed as

42 $$zT = \frac{{\sigma S^{2} }}{{\kappa_{e} + \kappa_{l} }}T = \frac{{ne\mu S^{2} }}{{ne\mu LT + \kappa_{l} }}T.$$

With the above derivation for *n*, *μ*, *S*, and *L*, we can rewrite *zT* as

43 $$\begin{aligned} zT & = \frac{{\frac{{2\hbar C_{l} N_{v} k_{B}^{2} T}}{{\pi m_{I}^{*} E_{def}^{2} }} {^{0} F_{ - 2}^{1}} \left( {\frac{{ ^{1} F_{ - 2}^{1} }}{{ ^{0} F_{ - 2}^{1} }} - \eta } \right)^{2} }}{{\frac{{2\hbar C_{l} N_{v} k_{B}^{2} T}}{{\pi m_{I}^{*} E_{def}^{2} }} {^{0} F_{ - 2}^{1}} \left[ {\frac{{ ^{2} F_{ - 2}^{1} }}{{ ^{0} F_{ - 2}^{1} }} - \left( {\frac{{ ^{1} F_{ - 2}^{1} }}{{ ^{0} F_{ - 2}^{1} }}} \right)^{2} } \right] + \kappa_{l} }}T \\ & = \frac{{\left( {\frac{{ ^{1} F_{ - 2}^{1} }}{{ ^{0} F_{ - 2}^{1} }} - \eta } \right)^{2} }}{{\left[ {\frac{{ ^{2} F_{ - 2}^{1} }}{{ ^{0} F_{ - 2}^{1} }} - \left( {\frac{{ ^{1} F_{ - 2}^{1} }}{{ ^{0} F_{ - 2}^{1} }}} \right)^{2} } \right] + \frac{{\pi m_{I}^{*} E_{def}^{2} }}{{2\hbar C_{l} N_{v} k_{B}^{2} T}}\frac{1}{{ ^{0} F_{ - 2}^{1} }}\kappa_{l} }} \\ & = \frac{{\left( {\frac{{ ^{1} F_{ - 2}^{1} }}{{ ^{0} F_{ - 2}^{1} }} - \eta } \right)^{2} }}{{\left[ {\frac{{ ^{2} F_{ - 2}^{1} }}{{ ^{0} F_{ - 2}^{1} }} - \left( {\frac{{ ^{1} F_{ - 2}^{1} }}{{ ^{0} F_{ - 2}^{1} }}} \right)^{2} } \right] + \frac{1}{{B ^{0} F_{ - 2}^{1} }}}}, \\ \end{aligned}$$

where *B* is a constant and is the quality factor. According to the position of *B* in the formula, one can readily see that large *B* leads to high *zT* values. The quality factor reported by literatures is usually given by^4,6,14^

44 $$\beta = \frac{{2k_{B}^{2} T\hbar C_{l} N_{v} }}{{3\pi m_{I}^{*} E_{def}^{2} \kappa_{l} }} = \frac{1}{3}B,$$

which is one third of *B*. Despite of that, the quality factor is a scale to estimate the performance of thermoelectric materials. In our SKBcal coding, we adopt *β* as the quality factor for consistency with literatures.

## Code implementation

### Generalized Fermi–Dirac integral (GFDI) within the framework of SKB model

According to the expressions of transport parameters, the GFDIs of $^{0} F_{ - 2}^{1}$, $^{1} F_{ - 2}^{1}$, $^{2} F_{ - 2}^{1}$, $^{0} F_{ - 4}^{1/2}$, and $^{0} F_{0}^{3/2}$should be calculated. However, it is a difficult task to obtain these values by direct integration. Hence, we decide to solve the GFDIs by a simple numerical method of Riemann integral. In the beginning, the differentiation of the Fermi distribution is derived as

45 $$- \frac{\partial f}{{\partial x}} = - \frac{\partial }{\partial x}\left( {\frac{1}{{e^{x - \eta } + 1}}} \right) = \frac{{e^{x - \eta } }}{{\left( {e^{x - \eta } + 1} \right)^{2} }}.$$

**Figure 1:**
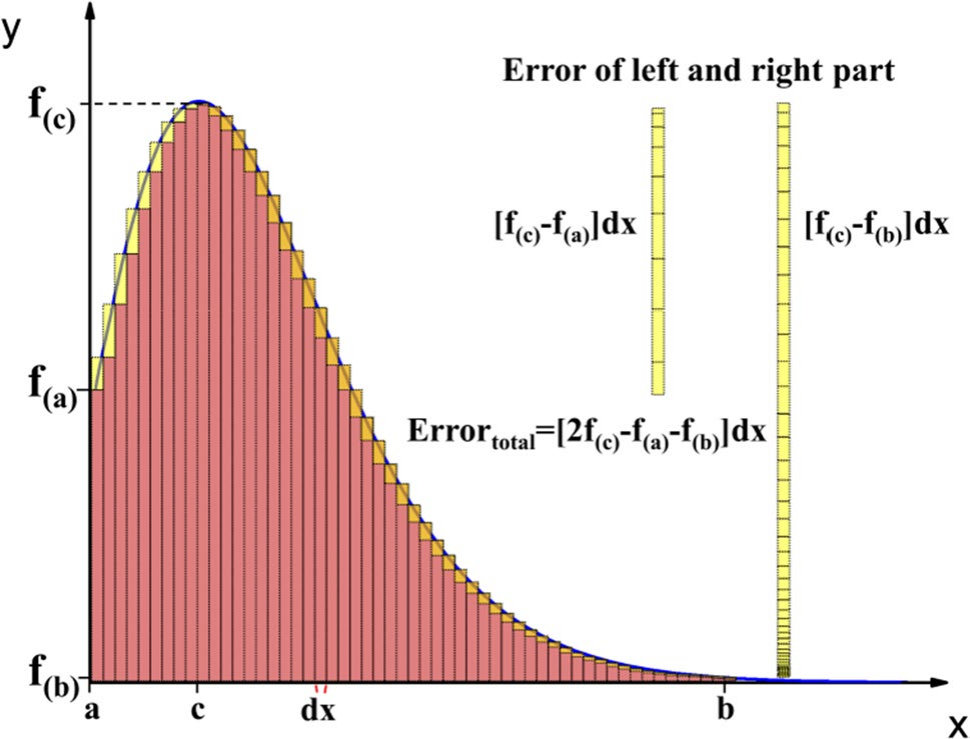
The illustration for analyzing the error of left Riemann integral method.

By substituting Eq. (44) into Eq. (3), the GFDI can be expressed as

46 $$^{n} F_{k}^{m} = \mathop \int \limits_{0}^{\infty } \frac{{e^{x - \eta } }}{{\left( {e^{x - \eta } + 1} \right)^{2} }}x^{n} \left( {x + \varepsilon x^{2} } \right)^{m} \left[ {\left( {1 + 2\varepsilon x} \right)^{2} + 2} \right]^{k/2} dx,$$

where *η* is the reduced Fermi level. It is well known that the size of regular partition (*dx*) would affect the accuracy of the resulting value in the Riemann method. In order to get the idea of their correlation, we calculate the significant digits of relative error (SDORE) for each GFDI using different partition sizes. The relative error is obtained via dividing absolute error by GFDI integration value. The absolute error is usually estimated using the mean value theorem and is given by

47 $$E_{Riemann} \le \frac{{f_{\left( b \right)} - f_{\left( a \right)} }}{b - a}\frac{{\left( {b - a} \right)^{2} }}{p} \le M\frac{{\left( {b - a} \right)^{2} }}{p},$$

where *M* is the greatest first derivative over the interval [a, b], *p* the magnitude of regular partition, and *f*_*(x)*_ represents the function of each GFDI. However, the way of predicting the maximum error is not quite suitable to GFDIs within the framework of the SKB model. The slope on the curve of *f*_*(x)*_ is sometimes very large and even becomes infinite. Therefore, we adopt a simple method to estimate the error. As seen in Fig. 1, we separate the error into left and right parts when an integral is calculated by the left Riemann integral method. The sum of the left part is *[f*_*(c)*_ *−* *f*_*(a)*_*]dx*, where *f*_*(c)*_ is the maximum and *f*_*(a)*_ the initial value of the function on the interval [a, b]. On the other hand, the sum of right part is *[f*_*(c)*_ *−* *f*_*(b)*_*]dx*, where *f*_*(b)*_ is the final value of the function. Thus, the total error of the left Riemann method can be expressed as

48 $$E_{total} \le \left[ {2f_{\left( c \right)} - f_{\left( b \right)} - f_{\left( a \right)} } \right]dx.$$

The predicted error using the above method is better than that estimated by the mean value theorem because of the slope problem of GFDIs in the SKB model.

**Figure 2:**
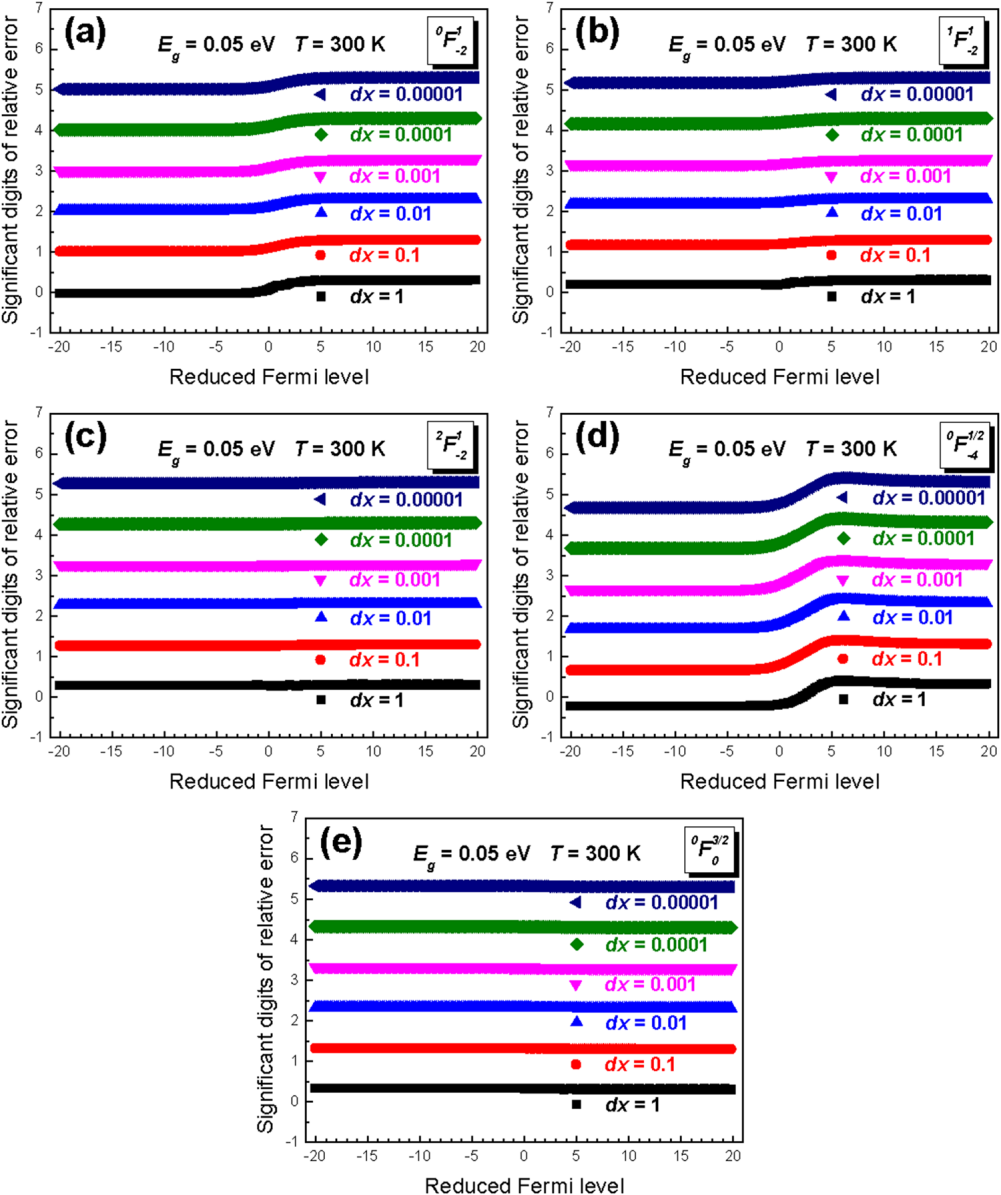
The significant digits of relative error (SDORE) of GFDIs with [n, m, k] of (**a**) [0, 1, -2], (**b**) [1, 1, -2], (**c**) [2, 1, -2], (**d**) [0, 0.5, − 4], and (**e**) [0, 1.5, 0] in different size of regular partition from 1 to 0.00001.

In order to get the idea of the influence of regular partition on the error, we calculate the significant digits of relative error (SDORE) of each GFDI using different partition sizes. The SDORE in this study is given by

49 $$SDORE = - \log_{10} \left( {\frac{{E_{total} }}{GFDI\; integration\; value}} \right),$$

which shows the level of accuracy in calculations. As seen in Fig. 2, the SDORE increases significantly with smaller regular partition. For simplicity, the band gap and absolute temperature are set as 0.05 eV and 300 K, respectively. The SDORE of each GFDI are almost at a similar level in the same regular partition. Overall, the SDORE shows little dependence of on the reduced Fermi level. However, the SDORE for $^{0} F_{ - 2}^{1}$and $^{0} F_{ - 4}^{1/2}$does show some increases when the reduced Fermi level turns from negative to positive. It is worth mentioning that the SDORE is about 4 if the regular partition is 0.0001, this accuracy is sufficient to perform the calculation. Hence, we would select this partition size of 0.0001 for calculating all the transport parameters.

**Figure 3:**
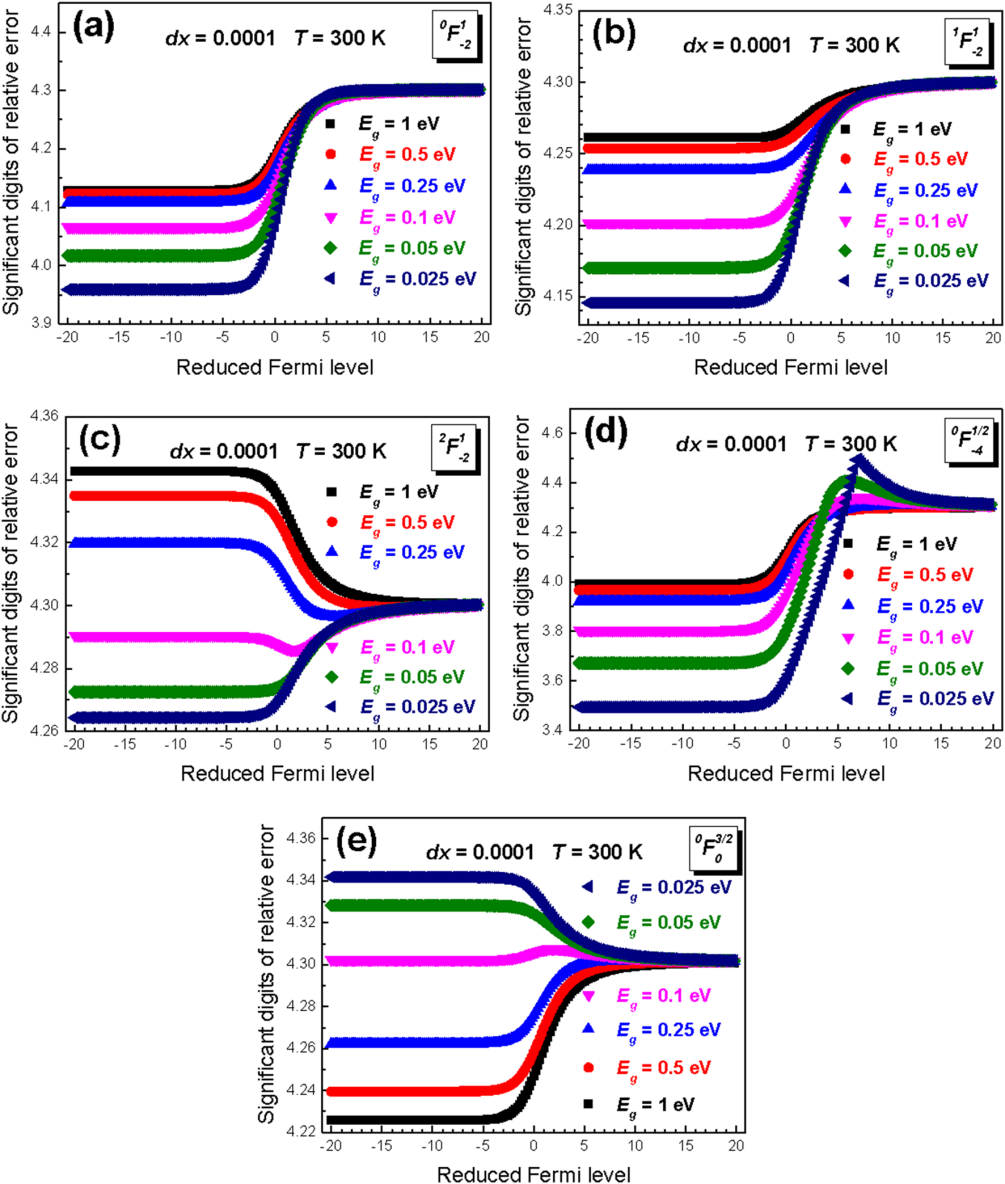
The significant digits of relative error (SDORE) of GFDIs with [n, m, k] of (**a**) [0, 1, -2], (**b**) [1, 1, -2], (**c**) [2, 1, -2], (**d**) [0, 0.5, − 4], and (**e**) [0, 1.5, 0] in different magnitude of energy band gap from 0.025 to 1 eV.

For a complete analysis, we calculate the SDORE using different magnitudes of band gap and absolute temperature. Figure 3 shows the reduced Fermi level dependence of SDORE for different values of band gap ranging from 0.025 to 1 eV. Even though the SDORE differs in various band gaps, it is relatively small and approaches zero at high reduced Fermi level.

### Reduced Fermi level (η)

In this section, we would discuss the relation between the thermopower and reduced Fermi level. The interval of the reduced Fermi level should be determined in the calculation. The reduced Fermi level can be refined and determined by experimentally measured thermopower using Eq. (1), which is rewritten as

50 $$\eta = \pm \left( {\frac{{ ^{1} F_{ - 2}^{1} }}{{ ^{0} F_{ - 2}^{1} }} - \frac{eS}{{k_{B} }}} \right).$$

**Figure 4:**
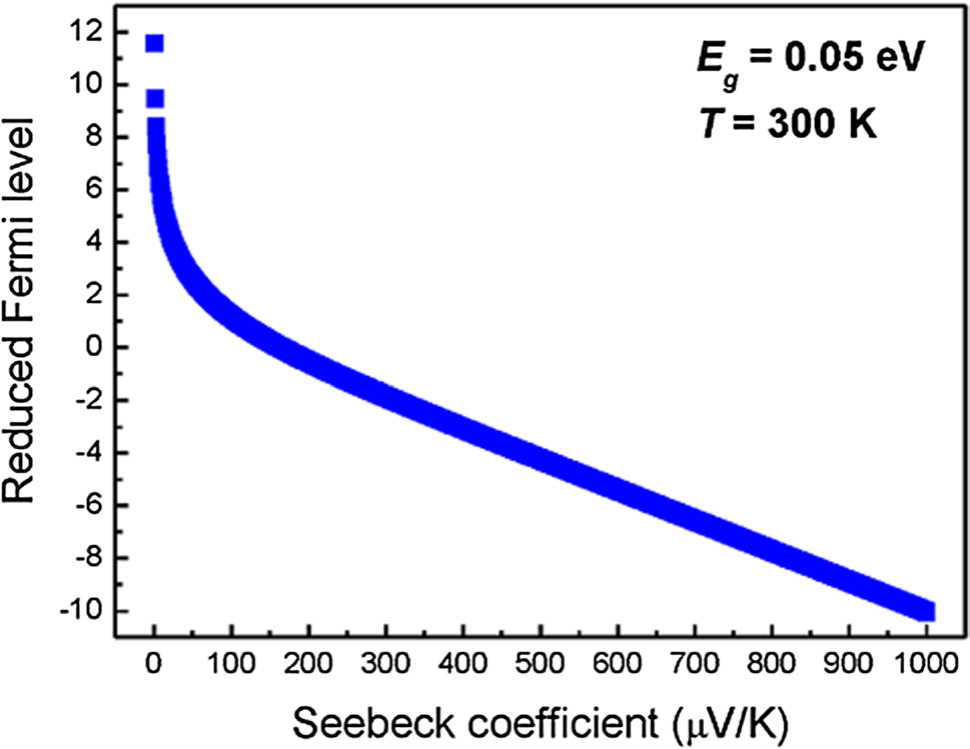
The Seebeck coefficient (thermopower) dependence of reduced Fermi level with the parameters of *dx* = 0.0001, *Eg* = 0.05 eV, and *T* = 300 K.

In the refinement, the method of iteration is adopted to obtain the refined reduced Fermi level since the reduced Fermi level involves with GFDIs. In order to confirm the suitable upper limit in GFDIs, the reduced Fermi level dependence of thermopower is calculated and plotted, which is shown in Fig. 4. We take the regular partition of 0.0001, the band gap of 0.05 eV, and the temperature of 300 K for the calculation. It can be readily seen that all the reduced Fermi level is less than 12 for thermopower in the range of 1 to 1000 μV/K. It is noted that materials with a small thermopower less than 10 μV/K can be viewed as metals, which are not suitable for calculation using the SKB model. However, the interval of [0, 80] is chosen in our calculation using SKBcal. Furthermore, the reduced Fermi level is about 56.1288 under the calculation conditions: thermopower = 0, partition size = 0.0001, band gap = 0.05 eV, and T = 300 K. However, we will not consider the case of thermopower = 0 in SKBcal, in particular for thermoelectric materials.

In the coding, we use the functions of *"For"* and *"While"* to build the combination of iteration. After entering the experimentally determined value of thermopower in SKBcal, a test value of zero is assigned to the initial reduced Fermi level, which is used to plugged into GFDIs. The values of GFDI integration are easily calculated by *"For"* loop in SKBcal. A rough reduced Fermi level is then obtained by Eq. (50). The reduced Fermi level is further refined by the *While"* loop. The reduced Fermi level is determined and recorded and saved in the file once the absolute value of the difference between the rough and calculated reduced Fermi level smaller than 0.0001, namely, *"abs(RFL-testRFL)* in the *"While"* loop meets the criteria of *"abs(RFL-testRFL)* < *0.0001"*. As such we can refine the reduced Fermi level to the fourth decimal place. It should be noted in our SKBcal coding that the regular partition size is set to 0.01 for the initial guess of the rough value of reduced Fermi level to speed up the runtime of iteration. After the rough value is obtained, the regular partition size is set to 0.0001 for the subsequent refinement of the reduced Fermi level and subsequent calculation of transport parameters.

### Lorenz number (L), electronic thermal conductivity (κ_e_), and lattice thermal conductivity (κ_l_)

After the refined reduced Fermi level is obtained, the Lorenz number is then calculated immediately by Eq. (2). Likewise, the electronic thermal conductivity is estimated by the Wiedemann–Franz law using Eq. (4). The lattice thermal conductivity is then computed by subtracting the electronic thermal conductivity from the measured total thermal conductivity.

### Hall factor (A), carrier concentration (n), carrier mobility (μ), and effective mass (m^*^)

Utilizing the SKB model, the electronic transport parameters including the Hall factor, carrier concentration, and density-of-states effective mass at the band edge can be calculated by using Eqs. (8), (10), and (11), respectively. Besides, the carrier mobility is given by

51 $$\mu = \frac{{\mu_{H} }}{A}.$$

The ratio of the density-of-states effective mass at the band edge relative to the rest electron mass is directly calculated in the SKBcal.

### Quality factor (β) and calculated thermoelectric figure of merit (zT)

Calculating and predicting the *zT* is one of the important functions in our SKBcal, which is beneficial to optimizing the thermoelectric properties of a material. However, the quality factor *β* is one of the parameters in the theoretical *zT* calculation. As seen in Eq. (16), *β* involves the elastic moduli (*C*_*l*_), deformation potential coefficient (*E*_*def*_), and the inertial mass (*m*_I_^*^). In general, there is difficulty in finding these three material parameters from literatures. However, we find an easy way to obtain *β* from the carrier mobility. By comparing Eqs. (12), (16), and (50), the expression of *β* can be rewritten as

52 $$\beta = \mu \frac{{k_{B}^{2} T\left( {2m_{0}^{*} k_{B} T} \right)^{3/2} }}{{9\pi^{2} \hbar^{3} e\kappa_{L} }}\frac{{ ^{0} F_{0}^{3/2} }}{{ ^{0} F_{ - 2}^{1} }}.$$

where the density-of-states effective mass at the band edge and the lattice thermal conductivity can be obtained from the previous section. As such we can calculate *β* and *zT* without knowing the values of *C*_*l*_, *E*_*def*_, and *m*_I_^*^ in Eq. (16).

## Code availability

The algorithm for calculating the transport properties of thermoelectric materials can be accessed through the Supplementary Information.

## Data verification

To confirm the accuracy of our SKBcal coding, we extract electrical resistivity (*ρ*), thermopower (*S*), and Hall concentration (*n*_*H*_) from graph data in Ref.^11^ using WebPlotDigitizer^23^. We then calculate the density-of-states effective mass using our algorithm of SKBcal by setting the value of the band gap, band degeneracy, and *K* being 0.43 eV, 1, and 1, respectively. Table 1 shows the comparison between our calculated results (shaded area in the table) of density-of-states effective mass and the lattice thermal conductivity (*κ*_*l*_) and those calculated based on the SKB model in Ref.^11^. The percent error shown in parenthesis is given by

53 $$\frac{{\left[ {\left( {\text{literature value}} \right) - \left( {\text{calculated value}} \right)} \right]}}{{\left( {\text{literature value}} \right)}} \times 100{\text{\% }}$$

**Table 1:** Comparison between the parameters captured from ref.^11^ and the parameters calculated using our SKBcal. The values with gray background are calculated using our SKBcal. The bracketed percentages is the error given by [(captured value) − (calculated value)]/(captured value) × 100%.

Ref.^11^	Cu_2+δ_SnSe_4_ (δ = − 0.01)				
*T* (K)	*ρ* (mΩ cm)	*S* (μV/K)	*n*_*H*_ (10^18^ cm^−3^)	*m*_0_* (*m*_*e*_)	*κ*_*l*_ (W/mK)
350	55.75	342.3	3.646	0.9659	1.606
				0.9523 (1.41%)	1.602 (0.249%)
400	67.15	354.4	4.327	1.064	1.332
				1.031 (3.10%)	1.374 (3.15%)
425	70.43	367.3	4.894	1.214	1.219
				1.169 (3.71%)	None
525	37.28	395.7	14.71	2.515	0.8894
				2.484 (1.23%)	None
550	33.02	370.7	22.28	2.622	0.8341
				2.573 (1.87%)	0.8369 (0.336%)
600	26.94	366.0	33.96	3.012	0.7434
				3.023 (0.365%)	0.7257 (2.38%)

The percent error in Table 1 is lower than 4%, which should be acceptable because transport parameters used in calculation show temperature discrepancy due to different experiments (resistivity, thermopower, and Hall effect). Besides, there are two cells entered as "None" because the total thermal conductivities at 425 and 525 K are not recorded in Ref.^11^.

To further verify the SKBcal, we repeat the same procedure as above and make a comparison between calculated data obtained using SKBcal with one more set of data reported in Ref.^24^, and show the results in Table 2. The value of the band gap, band degeneracy, and *K* is set to 0.39 eV, 1, and 1, respectively. Below the temperature of 600 K, the percent error is lower or near 5%. However, the percent error of the lattice thermal conductivity suddenly increases to 15% at 700 K. This large percent error can be ascribed to the phase transition of CdSnAs_2_ at high temperatures^25^. According to both of the slopes in *dρ*/*dT* and *dS/dT* change around 600 K reported in Ref.^24^. This indicates that the electronic structure of Cd_0.95_Zn_0.05_SnAs_2_ around that temperature has turned to a different state from room temperature. Therefore, the band gap of 0.39 eV adopted in calculation below 600 K is no longer good for T ≥ 600 K.

**Table 2:** Comparison between the parameters captured from Ref.^24^ and the parameters calculated using our SKBcal. The values with gray background are calculated using our SKBcal.

Ref.^24^	Cd_0.95_Zn_0.05_SnAs_2_				
*T* (K)	*ρ* (mΩ cm)	*S* (μV/K)	*n*_*H*_ (10^18^ cm^−3^)	*m*_0_* (*m*_*e*_)	*κ*_*l*_ (W/mK)
300	0.7810	− 47.75	2.260	0.03954	5.179
				0.03877 (1.95%)	5.235 (1.08%)
400	0.8208	− 71.77	2.252	0.04514	3.648
				0.04488 (0.576%)	3.697 (1.34%)
500	0.8615	− 86.59	2.240	0.04717	2.689
				0.04480 (5.02%)	2.757 (2.53%)
600	0.8995	− 103.8	2.252	0.04943	2.089
				0.04728 (4.35%)	2.312 (10.7%)
700	0.9014	− 108.6	2.417	0.04771	1.609
				0.04560 (4.42%)	1.861 (15.7%)

Table 3 shows the comparison between calculated results using SKBcal and transport parameters reported in Ref.^26^. The transport parameters of Lorenz number (*L*), electronic thermal conductivity (*κ*_*e*_), and lattice thermal conductivity (*κ*_*l*_) in Ref.^26^ are calculated by Mathematica using Riemann integral method. As compared to the results obtained using our SKBcal by Python, the difference between them is almost negligible.

**Table 3:** Comparison between the parameters captured from Ref.^25^ and the parameters calculated using our SKBcal. The values with gray background are calculated using our SKBcal.

Ref.^25^	Ag_2_Te–Ag composite				
*T* (K)	*ρ* (mΩ cm)	*S* (μV/K)	*L* (10^–8^ WΩ/K^2^)	*κ*_*e*_ (W/mK)	*κ*_*l*_ (W/mK)
300	1.074	− 88.94	1.377	0.3845	0.3564
			1.377	0.3845	0.3564
350	0.8652	− 85.08	1.370	0.5540	0.2426
			1.369	0.5540	0.2426
400	0.7851	74.34	1.410	0.7185	0.2463
			1.410	0.7184	0.2464
525	2.091	− 112.03	1.456	0.3656	0.2375
			1.456	0.3656	0.2375
573	2.155	− 122.98	1.397	0.3713	0.2024
			1.397	0.3715	0.2022

## Conclusion

We develop an algorithm called SKBcal coded by Python 3.7 to calculate and predict the transport parameters for materials with a single nonparabolic band structure within the framework of the SKB model. The generalized Fermi–Dirac integrals (GFDIs) in this study are calculated by the left Riemann integral method. To quickly obtain the reduced Fermi level, the iteration process is implemented by two stages: the first stage to obtain a rough value, which is then used at the 2nd stage for determining the refined reduced Fermi level. The determined reduced Fermi level is then used for subsequent calculation of all transport parameters. Accuracy of numerical integration is determined by the significant digits of relative error (SDORE) which is larger than 4 when setting the regular partition smaller than 0.0001. As compared to the data reported in the literature, the results calculated using SKBcal are in good agreement with the data reported by literature.

## Supplementary Information

Supplementary Information.

## References

[1] Chang K-C, Liu C-J (2020). An algorithm of calculating transport parameters of thermoelectric materials using single band model with optimized integration methods. Comput. Phys. Commun..

[2] Kane EO (1957). Band structure of indium antimonide. J. Chem. Solids..

[3] Ravich YuI, Efimova BA, Smirnov IA (1970). Semiconducting Lead Chalcogenide.

[4] Pei Y, LaLonde AD, Wang H, Snyder GJ (2012). Low effective mass leading to high thermoelectric performance. Energy Environ. Sci..

[5] Lee H (2016). Thermoelectrics Design and Materials.

[6] Pei Y, Wang H, Gibbs ZM, LaLonde AD, Snyder GJ (2012). Thermopower enhancement in Pb_1−x_Mn_x_Te alloys and its effect on thermoelectric efficiency. NPG Asia Mater..

[7] Lin S, Li W, Zhang X, Li J, Chen Z, Pei Y (2017). Sb induces both doping and precipitation for improving the thermoelectric performance of elemental Te. Inorg. Chem. Front..

[8] Herring C, Vogt E (1956). Transport and deformation-potential theory for many-valley semiconductors with anisotropic scattering. Phys. Rev..

[9] Ren Z, Lan Y, Zhang Q (2019). Advanced Thermoelectrics Materials, Contacts, Devices, and Systems.

[10] Chasmar RP, Stratton R (1959). The thermoelectric figure of merit and its relation to thermoelectric generators. J. Electron. Contr..

[11] Li W, Lin S, Zhang X, Chen Z, Xu X, Pei Y (2016). Thermoelectric properties of Cu2SnSe4 with intrinsic vacancy. Chem. Mater..

[12] Zhou M, Gibbs ZM, Wang H, Han Y, Xin C, Li L, Snyder GJ (2014). Optimization of thermoelectric efficiency in SnTe: The case for the light band. Phys. Chem. Chem. Phys..

[13] Wu C-F, Wei T-R, Li J-F (2015). Electrical and thermal transport properties of Pb_1__−__x_ Sn_x_ Se solid solution thermoelectric materials. Phys. Chem. Chem. Phys..

[14] Wang H, Pei Y, LaLonde AD, Snyder GJ (2012). Weak electron–phonon coupling contributing to high thermoelectric performance in n-type PbSe. Proc. Natl. Acad. Sci. U.S.A..

[15] Wang H, Gibbs ZM, Takagiwa Y, Snyder GJ (2014). Tuning bands of PbSe for better thermoelectric efficiency. Energy Environ. Sci..

[16] He W, Wang D, Wu H, Xiao Y, Zhang Y, He D, Feng Y, Hao Y-J, Dong J-F, Chetty R, Hao L, Chen D, Qin J, Yang Q, Li X, Song J-M, Zhu Y, Xu W, Niu C, Li X, Wang G, Liu C, Ohta M, Pennycook SJ, He J, Li J-F, Zhao L-D (2019). High thermoelectric performance in low-cost SnS_0.91_Se_0.09_ crystals. Science.

[17] Xie, H. *et al.* The intrinsic disorder related alloy scattering in ZrNiSn half-Heusler thermoelectric materials. *Sci. Rep.* **4**, 6888 (2015). 10.1038/srep06888 PMC4217114 25363573

[18] Wang H, Schechtel E, Pei Y, Snyder GJ (2013). High thermoelectric efficiency of n-type PbS. Adv. Energy Mater..

[19] Zawadzki W, Kowalczyk R, Kolodziejczak J (1966). The generalized Fermi–Dirac Integrals. Phys. Stat. Sol..

[20] Zawadzki W, Szymanska W (1971). Electron scattering and transport phenomena in n-InSb. J. Phys. Chem. Solids.

[21] Kolodziejczak J, Zukotynski S (1964). Electron scattering and transport phenomena in n-InSb. Phys. Stat. Sol..

[22] Zawadzki W, Kolodziejczak J (1964). Transport properties of cubic semiconductors with nonparabolic energy bands. Phys. Stat. Sol..

[23] A. Rohatgi, *Web Plot Digitizer; 2015*. http://www.arohatgi.info/WebPlotDigitizer.

[24] Yao Z, Zhang X, Shen J, Chen B, Ang R, Li W (2019). Evaluation of thermoelectric CdSnAs2 with intrinsically low effective mass. J. Alloys Compd..

[25] Gasson DB, Holmes PJ, Jennings IC, Marathe BR, Parrott JE (1962). The properties of ZnSnAs2 and CdSnAs2. J. Phys. Chem. Solids.

[26] Lin F-H, Chang K-C, Yang Z-R, Gharleghi A, Liu C-J (2018). The effects of Ag nanoparticles on the thermoelectric properties of Ag2Te–Ag composite fabricated using an energy-saving route. J. Alloys Compd..

