# Supplementary material for: An algorithm of calculating transport parameters of thermoelectric materials using single Kane band model with Riemann integral methods
Source: Sci Rep. 2022 Apr 29;12:7056. doi: 10.1038/s41598-022-09734-4 (PMC9054765; doi:10.1038/s41598-022-09734-4)
Supplement: Supplementary file 1 — Supplementary Information. [file 41598_2022_9734_MOESM1_ESM.docx]

Code availability:

The algorithm for calculating the transport properties of thermoelectric materials can be accessed through the Supplementary Information.

***PROGRAM SUMMARY***

*Program title:* SKBcal

*Licensing provisions:* MIT license (MIT)

*Programming language:* Python 3.7

*Program obtainable from****:*** <https://github.com/ffalyye/SKBcal>

*Nature of program:* Calculating thermoelectric transport parameters for materials with a single nonparabolic band structure under assuming the acoustic phonon scattering as the major scattering mechanism.

*Methods:*

Calculating the generalized Fermi-Dirac integrals using the left Riemann integral method. Refining the reduced Fermi level by iteration using the combined function of *"For"* and *"While"*.
